# Maternal mental health services in Sri Lanka: challenges and solutions

**DOI:** 10.1192/bji.2020.52

**Published:** 2021-11

**Authors:** Aruni Hapangama, K.A.L.A. Kuruppuarachchi

**Affiliations:** 1 FRANZCP, Senior Lecturer, Department of Psychiatry, Faculty of Medicine, University of Kelaniya, Sri Lanka. Email: ahapangama@kln.ac.lk; 2 FRCPsych, Cadre Chair and Senior Professor of Psychiatry, Department of Psychiatry, Faculty of Medicine, University of Kelaniya, Sri Lanka

**Keywords:** Perinatal psychiatry, maternal mental health, antenatal depression, culture and stigma, postnatal depression

## Abstract

Sri Lanka boasts of making significant strides in the indicators of maternal healthcare during the past few decades. However, these indicators mostly look only at the physical well-being of women. Lack of awareness regarding maternal mental health problems among grass-root level healthcare workers, poor integration of services, and the culture and stigma regarding perinatal mental disorders are barriers to improving services in maternal mental healthcare in Sri Lanka.

Sri Lanka is an island situated in the Indian Ocean that gained its independence from the UK in 1948. It is slowly but steadily rebuilding itself from the devastations caused by more than three decades of ethnic conflict, the Indian Ocean tsunami on Boxing Day 2004 and the Easter Sunday bombings in 2019.

## Demography

Sri Lanka was recently categorised as a country with an upper-middle-income economy^1^ and the total population of the country in the year 2016 was around 21.2 million, with a gender ratio of 93.8 men per 100 women.^2^ Women in the reproductive age group (15–49 years) comprised 27.8% (5.6 million) of the total population of the country in 2016.^2^ The majority of the population were living in the rural sector (77%).^2^ Education indicators showed that 56.4% of the female population had completed education up to secondary level (i.e. up to 15 years of age).^2^ The female literacy rate was 94.5%.^2^ The mean age at marriage was 23.4 years for females.^3^ The total fertility rate was 2.4.^2^

Sri Lanka has a multi-ethnic society, with Buddhism, a religion that emphasises equal rights for women, being the religion of the majority^2^ of the population. The other religions practised among the people of Sri Lanka are Hinduism, Islam, Roman Catholicism and Christianity.^2^

## Changes in culture and society and their impact on women of childbearing age

Extensive social, political and economic changes since independence have led to significant changes in the role of women in Sri Lankan society.^4^ During most of the 20th century and to some extent even now, Sri Lankan women who become mothers have lived with their extended family.^4^

However, these traditions, including the extended family system and the obligation to live with ones’ inlaws, are gradually diminishing, especially in urban areas.^4^ Most couples value independent living apart from parents or inlaws. In addition, in current-day Sri Lanka, women have taken a leading role in the educational, social and political arenas.^4^ However, the fast dwindling support from the extended family, especially in urban settings, and their dual role as mothers and as members of the workforce may at times be stressful for women.

## General healthcare services in Sri Lanka

Healthcare in Sri Lanka is mainly provided by practitioners of Western medicine, although Ayurveda, Yunani, Sidda and homeopathy practitioners also contribute.^2^ The majority of services are provided free of charge by the government.^3^ The state health system provides 50% of medical treatment, 95% of hospital admissions and 99% of preventive care for the country.^3^

The primary healthcare services are centred around medical officers of health (MOHs), who are designated to cover well-defined areas. Several other primary healthcare practitioners, such as public health nursing sisters, public health inspectors and public health midwives, support the MOHs.

## Healthcare services for women of childbearing age

Combined maternal and child health services have been established in Sri Lanka since the 1920s. The maternal and child health (MCH) programme, which is primarily run by MOHs, concentrates on promoting antenatal care, delivery care and care for postpartum mothers.^3^ Antenatal care services in Sri Lanka cover 100% of the population and 95% of expectant females are registered for antenatal care before 12 weeks of pregnancy.^5^ About 85% of postnatal mothers are reported to receive at least one postnatal visit from a public health midwife during the first 10 days after their delivery.^5^ Around 94% of all deliveries take place in a public-sector health facilitiy.^6^ Urban areas report a higher proportion of births taking place in private health facilities (in rural areas, 100% of deliveries occur in public health facilities).^7^

There has been a huge decline in the maternal mortality ratio (MMR) in Sri Lanka, from 92 per 100 000 live births in 1990 to 36 per 100 000 in 2017.^8^ This decline is attributed to improved quality of obstetric care as well as timely referrals to hospitals by teams in the primary healthcare setting.^8^

## Mental health services for women in pregnancy and postpartum

Women who are found to have mental health problems during their pregnancy or postpartum are generally referred by an MOH, obstetrician or paediatrician to psychiatry services in either the state or private sector, depending on the severity of the problem and the preference of the woman and/or her family. The validated Sinhala translation of the Edinburgh Postnatal Depression Scale (EPDS)^9^ has been introduced into routine practice in MOH clinics, to screen for depression in women during the first 6 weeks postpartum. A few state and private sector hospitals located in more urban areas have dedicated mother and baby beds for women with postnatal mental illnesses.

## The burden of mental health problems among women during pregnancy and postpartum

Despite the relatively low overall MMR, the maternal mental health service remains largely a neglected area in Sri Lanka.^10^

A study carried out among antenatal women in a more rural area of Sri Lanka^10^ reported a prevalence of antenatal depression of 16.2%. This study, however, did not find significant associations with sociodemographic factors.

A large descriptive cross-sectional study^9^ reported the prevalence of postpartum depression as 27.1%. In this study primiparity, having had three or more pregnancies and a lower income level were found to be risk factors for developing postnatal depression.

Sri Lanka introduced the Maternal Death Surveillance and Response (MDSR) system in 1981, where deaths by suicide up to 42 days postpartum are reviewed by a team led by a consultant psychiatrist using a psychological autopsy tool, which helps to translate findings into policies.^8^ It is reported that the rate of maternal suicide has increased from 0.8 per 100 000 live births in 2002 to 12.1 per 100 000 live births in 2010.^11^ One explanation for these alarming figures may be the improvement in the health information system in recording the cause of death. However, figures such as these cause significant concern as they highlight the need for identification and addressing of factors associated with maternal suicide. A study conducted in a rural district of Sri Lanka reported that 17.8% of recorded maternal deaths were due to suicide and 79% of the women who had killed themselves were less than 30 years old.^12^

## Challenges in the development of services

Policy documents on maternal and child health and documents by various funding agencies in Sri Lanka^5^ do not appear to give much priority to the assessment and management of perinatal mental illnesses.

Despite being shown to be effective in screening for antenatal depression, the EPDS is still being used only to screen for postnatal depression in the Sri Lankan setting. A Tamil (the second most spoken language on the island) version of the EPDS is not yet available.

Only a few secondary and tertiary care hospitals have dedicated mother and baby beds for women with postnatal psychiatric disorders. This lack of facilities at times results in the mother being admitted to a general adult psychiatry ward to receive the required treatment during the postpartum period while the newborn is either admitted to a paediatric ward with a family member or sent home.

Low identification of these illnesses due to lack of awareness among primary healthcare workers, as well as poor integration between maternal health services and mental health services, may also play a role in the relatively high prevalence of perinatal psychiatric disorders and maternal suicides.

In Sri Lanka, culture and stigma may also play a part in the underdiagnosis and undertreatment of perinatal mental illnesses. Demonological and astrological remedies have at times been commonly used in the treatment of postpartum mental illnesses and may lead to delays in women receiving effective treatment.

There is a significant gap in knowledge regarding the psychological effects of the three decades of ethnic conflict, the Boxing Day tsunami of 2004 and domestic violence on women in the perinatal period.

## Ways to improve services

Educating women regarding mental health problems and their management should be incorporated into the already well-established MCH programme. Screening and management of maternal mental health problems should be given more prominence in national health policies.

All primary healthcare practitioners and grass-roots level healthcare workers need to undergo regular training in basic screening, assessment and management of perinatal mental health problems. The Sri Lanka College of Psychiatrists could partner up with the Ministry of Health and other stake holders to conduct such programmes.

Another area that needs urgent attention is the unequal distribution of services in some districts of Sri Lanka for these women. The Ministry of Health and the relevant colleges should take a more active role in addressing this.

Women with mental health problems in the perinatal period should be educated about their condition and offered continuity of care with evidence-based interventions.

Integration and coordination between the MCH programme and mental health teams in hospitals in detecting, referring, treating and following up of women with perinatal mental disorders should be further strengthened by improving communications between these teams.

Nationwide research into prevalence and service needs should be carried out to formulate and influence policies.

Psychiatrists need to take a more active role in combating cultural beliefs and stigma among patients and their families by introducing maternal mental health literacy among the general public and advocating for policies to improve services for women in the perinatal period.

## Conclusions

The timely detection and treatment of perinatal mental illnesses are of utmost importance. It is essential to educate policy makers and professionals regarding these and to develop innovative research and policies that are culturally sensitive, feasible as well as sustainable.

## Supplementary material

For supplementary material accompanying this paper visit https://doi.org/10.1192/bji.2020.52. click here to view supplementary material
